# A new antisarcoma strategy: multisubtype heat shock protein/peptide immunotherapy combined with PD-L1 immunological checkpoint inhibitors

**DOI:** 10.1007/s12094-021-02570-4

**Published:** 2021-04-01

**Authors:** H. Li, X. Sui, Z. Wang, H. Fu, Z. Wang, M. Yuan, S. Liu, G. Wang, Q. Guo

**Affiliations:** 1 Institute of Orthopedics, Chinese PLA General Hospital, Beijing Key Laboratory of Regenerative Medicine in Orthopedics, Key Laboratory of Musculoskeletal Trauma War Injuries, PLA, No. 28 Fuxing Road, Haidian District, Beijing, 100853 China; 2 Changzhi Second People’s Hospital, Changzhi, 046000 Shanxi China; 3 School of Medicine, Nankai University, Tianjin, 300071 China; 4 Department of Microbiology and Immunology, Shanxi Medical University, Taiyuan, 030001 Shanxi China

**Keywords:** Osteosarcoma, mHSP/peptide sarcoma vaccine, Grp94/peptide sarcoma vaccine, PD-L1, IFN-γ

## Abstract

Osteosarcoma, a common malignant tumor in orthopedics, often has a very poor prognosis after lung metastasis. Immunotherapy has not achieved much progress in the treatment because of the characteristics of solid tumors and immune environment of osteosarcoma. The tumor environment is rather essential for sarcoma treatment. Our previous study demonstrated that heat shock proteins could be used as antitumor vaccines by carrying tumor antigen peptides, and we hypothesize that an anti-osteosarcoma effect may be increased with an immune check point inhibitor (PD-L1 inhibitor) as a combination treatment strategy. The present study prepared a multisubtype mixed heat shock protein osteosarcoma vaccine (mHSP/peptide vaccine) and concluded that the mHSP/peptide vaccine was more effective than a single subtype heat shock protein, like Grp94. Therefore, we used the mHSP/peptide vaccine in combination with a PD-L1 inhibitor to treat osteosarcoma, and the deterioration of osteosarcoma was effectively hampered. The mechanism of combined therapy was investigated, and AKT expression participates with sarcoma lung metastasis. This study proposed an antisarcoma strategy via stimulation of the immune system as a further alternative approach for sarcoma treatment and elucidated the mechanism of combined therapy.

## Introduction

Osteosarcoma (OS) often occurs in long bones, including the distal femur, proximal humerus and distal humerus, as one of the most common malignant cancers in orthopedics, and children and adolescents are the main disease groups [1, 2]. The overall prognosis is poor. The combination of surgery and chemotherapy is the current main treatment strategy. However, resistance to chemotherapy often occurs during treatment and leads to tumor recurrence [2]. Tumor immunotherapy had enormous success in leukemia, and it is expected to become a new treatment for osteosarcoma. Heat shock proteins (HSPs) are one of the most abundant protein families in cells and are involved in various biological activities [3]. HSPs are found primarily in the cytoplasm, endoplasmic reticulum (ER), cell surface and extracellular matrix [4–7]. HSPs play an important role in antigen uptake and may function as molecular chaperones in the construction of immune defense systems [8–10]. The mechanism of HSP protein vaccine was described. The major cell surface receptor that binds to the HSP/peptide complex is CD91, which is internalized via receptor-mediated endocytosis and presented on the cell surface in the presence of MHC class I molecules to stimulate NK cell activation [11–14]. Russia performed clinical trials for Grp94/peptide and HSP70. Their experiments found that HSPs played a key role in the development of antitumor responses by acting as carriers for tumor-derived immunogenic peptides, as adjuvants for antigen presentation, or as targets for the innate immune system [15, 16]. Our previous studies [17, 18] prepared a mixed multisubtype HSP (mHSP) vaccine and revealed an antitumor effect. We hypothesized that mHSP/peptides would be more effective than a single subtype protein vaccine, and the present study compared the antisarcoma effect of mHSP/peptide and the single subtype Grp94/peptide.

Immune escape is an urgent problem in cancer treatment. A better understanding of the mechanisms of immune escape is crucial for the development of strong anticancer strategies [19]. The inhibitory factor PD-L1 is expressed on cancer cells, and it is closely related to the poor prognosis of tumors [20]. PD-L1 transmits a signal that inhibits lymphocyte activation via binding to the PD-1 receptor on lymphocytes [21]. The upregulation of IFN-γ induces PD-L1 expression on tumor cells. The upregulation of secreted IFN-γ induced PD-L1 expression in a mouse melanoma model [22, 23].

Combination immunotherapy for the treatment of tumors is a current cancer treatment method. The present study prepared an mHSP/peptide vaccine and Grp94/peptide vaccine to compared their antisarcoma abilities. We tested the antitumor effects of combination therapy of the mHSP/peptide vaccine plus PD-L1 immunological checkpoint inhibitors.

## Materials and methods

### Mice, cell lines, and reagents

Female C57BL/6 mice were purchased from Beijing SPF (Beijing) Biotechnology Co., Ltd. Mice, from 6 to 8 weeks of age and weighing 18–22 g, were selected for all experiments. All mice were reared at the Animal Laboratory of the Fifth Medical Center of the General Hospital of the People’s Liberation Army and were raised according to specific pathogen-free (SPF)-class rodent feeding methods and conditions. Animal experiments were performed strictly in accordance with the Guide for the Management and Use of Laboratory Animals issued by the National Institutes of Health.

The fibroblast cell line (L929) was provided by the Institute of Orthopedics of the Chinese People’s Liberation Army General Hospital. Mouse fibrosarcoma cells (MCA207) were provided by Professor Zong Kangla from Stanford University. All cell lines were cultured in DMEM supplemented with 10% fetal bovine serum (FBS), 100 units/mL penicillin G, and 100 μg/mL streptomycin sulfate. DMEM, FBS and penicillin G were acquired from Gibco Laboratories (Grand Island, NY, USA). Recombinant mouse IFN-γ was purchased from PEPRO TECH. Monoclonal antibodies (mAbs) against mouse HSP70, HSP90, Grp94, PD-L1, CD4, CD8 and FOXP3 were purchased from Abcam. The flow cytometry anti-mouse PD-L1 (CD274) antibody was purchased from Bio-legend, Inc. The flow cytometry cytology kit and PD-L1 checkpoint inhibitor was purchased from Merck. The chromatographic columns included the Enrich SEC650 molecular sieve column, UNOsphere Q anion exchange column and UNOsphere SUPrA affinity column, which were purchased from Bio-Rad.

### Immunohistochemistry

Immunohistochemical staining was used to identify HSPs in cells and tissue and quantify immune cells (CD4 + T cells, CD8 + T cells, and regulatory T cells (Tregs)). The following immunohistochemical staining process was used. Frozen slides were fixed with acetone or paraformaldehyde, and the slides were stored at − 4 °C. The slides were washed with PBS, and a 3% hydrogen peroxide solution was added for almost 5 min to inactivate horseradish peroxidase. The slides were washed again, and 2% sheep serum and 0.5% Triton were added for approximately 1–2 h. Primary antibodies against HSP70, HSP90, Grp94, PD-L1, CD4, CD8, and FOXP3 (concentration 1:200) were diluted in blocking solution (2% sheep serum) and allowed to stand overnight at 4 °C. The primary antibodies were removed, and HSP and CD4 + T cell staining were performed using fluorescence staining, and CD8 + T cell staining was performed using DAB. For fluorescence staining, a fluorescently labeled secondary antibody (concentration 1:100–1:200) was added for 1 h. DAPI staining (concentration 1:100; 5 min) was used to seal the slices, and an image was acquired under a confocal microscope. For DAB staining, 50 µL of MaxVision reagent was added to the primary antibody incubation for 10–15 min at room temperature then washed 3–5 times with PBS. In the last step, 100 µL of DAB solution was added to the slices, and hematoxylin was used to counterstain the nuclei.

### ELISA kits

Enzyme-linked immunosorbent assay (ELISA) kits for HSP 70, HSP 90, and Grp94 were purchased from RD Systems company, and ELISA kits for IFN-γ and TNF-α were purchased from MULTI Science Co., Ltd. The ELISA experiment was performed accurately according to the manufacturers’ instructions.

For HSP 70, HSP 90, and Grp94 detection, three mice from each group were killed at 14 days. MCA207 cells cultured in vitro and sarcoma tissue were disrupted using cell lyase then subjected to ELISA according to the manufacturers’ instructions. For IFN- γ, TNF-α, IL-2, and IL-10 detection, three mice from the different groups were killed at 14 and 28 days, and tumor tissues were collected to prepare a mixed protein suspension using the method described above. The experiments included determinations of IFN-γ, TNF-α, IL-2, and IL-10 concentrations, which were performed in accordance with the ELISA kit instructions (Lianke Bio).

### Preparation of mHSP/peptide and Grp94/peptide sarcoma vaccines

**Fig. 1:**
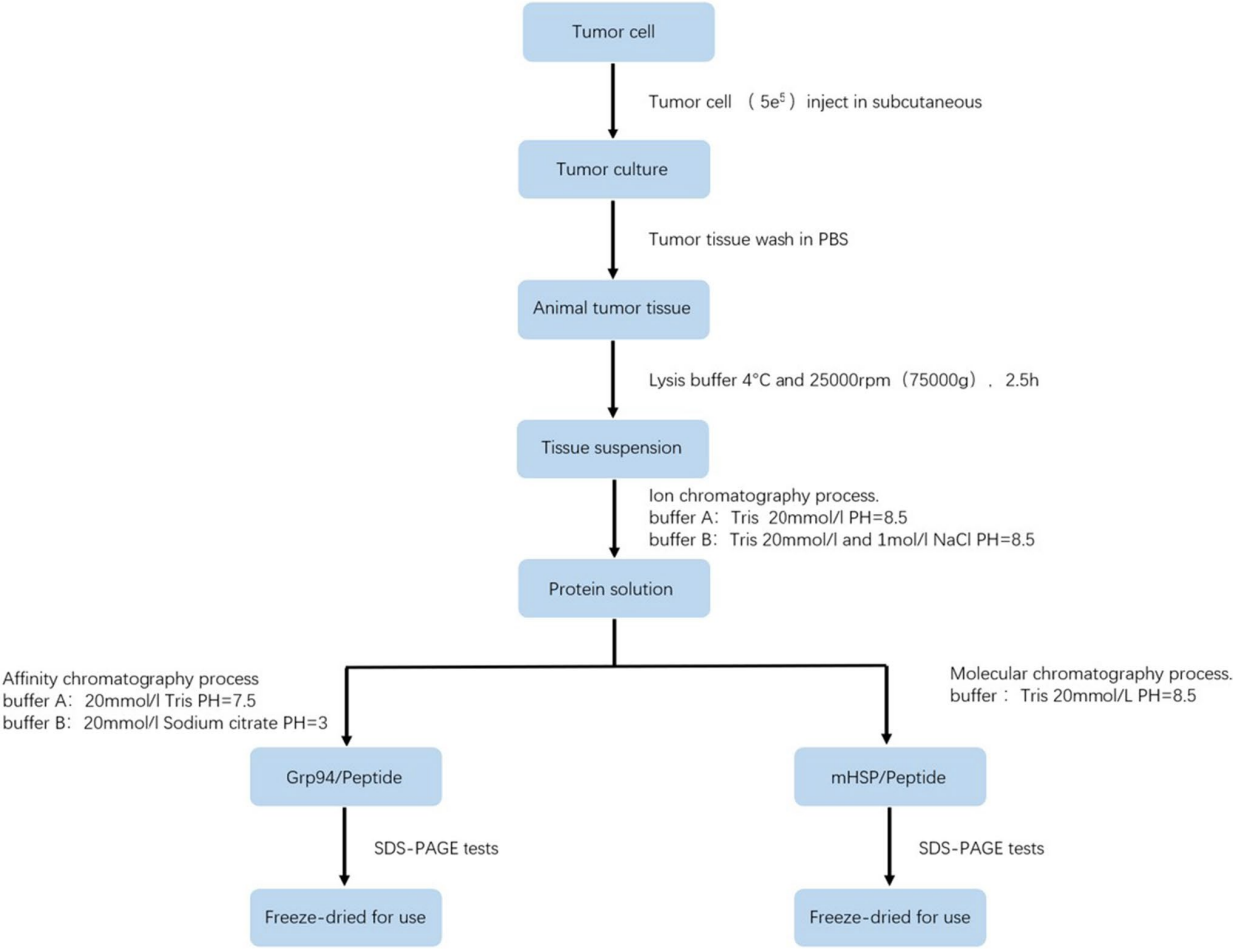
Flow chart of the mHSP/peptide vaccine and Grp94/peptide vaccine preparation

We prepared two different HSP vaccines, a Grp94/peptide vaccine and mHSP/peptide vaccine, for comparisons. Tumor tissue, which included Grp94 and mHSP, were obtained from tumor-bearing mice. The tumors grew in C57BL/6 mice that were subcutaneously injected with 5e5 MCA207 cells and separated after 3–4 weeks. The progress of vaccine extraction is described as follows. The tumor was minced and washed with PBS buffer with the addition of lysis buffer (RIPA:cocktail = 1:100, 4 °C) to extract tumor protein. The lysed tissue was subsequently placed in an ultracentrifuge (25,000 rpm, 75,000×*g*) for cell disruption and protein solubilization. The samples were collected and filtered. The samples were processed using an anion chromatography column (UNOsphere Q) with buffer (buffer A: 20 mmol/L Tris solution, pH = 8.5; buffer B: a mixed solution of 20 mmol/L Tris and 1 mol/L NaCl at pH = 8.5) to discard nonprotein substances. The mHSP/peptide and Grp94/peptide vaccines were prepared using different processes. The mHSP/peptide preparation process was performed in a molecular sieve chromatography column (Enrich SEC650) containing buffer (20 mmol/L Tris, pH = 8.5). The Grp94/peptide preparation was performed in an affinity immunochromatographic column (UNOsphere SUPrA) and buffer (buffer: A: 20 mmol/L Tris solution, pH = 7.5; buffer B: 20 mmol/L sodium citrate buffer, pH = 3; Grp94 antibody purchased from Abcam). The mHSP/peptide and Grp94/peptide were stored at − 80 °C under specific conditions (pH = 9) (Fig. 1).

### Western blot analysis

Protein was extracted from cell lysates prepared using RIPA lysate and a protease inhibitor. The amount of protein was determined using a BCA protein assay kit. Equal amounts of denatured protein were separated using sodium dodecyl sulfate–polyacrylamide gel electrophoresis (SDS-PAGE) and transferred to a polyvinylidene fluoride (PVDF) membrane that was blocked with 5% skim milk. Buffered PBS (TBS) containing 0.1% Tween-20 was used for washing. The following primary antibodies and concentrations were used: 1:1000 dilution of an anti-HSP70 antibody; 1:1000 dilution of an anti-HSP90 antibody; 1:1000 dilution of an anti-Grp94 antibody; and 1:1000 dilution of an anti-PD-L1 antibody. Membranes were incubated with a horseradish peroxidase (HRP)-conjugated secondary antibody. The reaction bands were detected using a Prime Western Blotting Detection System.

### Vaccine cytotoxicity assay

The cytotoxicities of the mHSP/peptide and Grp94/peptide were tested using flow cytometry. The mHSP/peptide vaccine was applied at four different concentrations to the fibroblast L929 cell line, and the same procedure was used for the Grp94/peptide vaccine. The four different concentrations of the mHSP/peptide vaccine ranged from 0 to 30 µg/mL, and the Grp94/peptide vaccine concentrations ranged from 0 to 300 ng/mL. The L929 cells were cultured in 6-well plates, and vaccines were added to 4 wells at different concentrations. The plates were placed in a carbon dioxide cell incubator for 24 h. A Muse flow cytometer (Merck Company) was used for the L929 viability tests. The flow cytometry test for L929 cell viability treated with different vaccine concentrations followed the instructions of the Muse Count/Viability manual.

### Animal model construction and animal vaccination experiment

Our study included three animal experiments. We constructed an animal model of sarcoma and produced the vaccines. We compared the antitumor effects of the Grp94/peptide vaccine and the mHSP/peptide vaccine. We tested and compared the antisarcoma effects of the combined method, i.e., a PD-L1 inhibitor plus mHSP/peptide, and single-therapy treatment. Mice were anesthetized with 0.1 mL 1% sodium pentobarbital to adhere to animal ethics guidelines. For model construction, MCA207 sarcoma cells were injected into the joint space of the mice (*n* = 5) using a 1-mL syringe at a cell concentration of 5e5/mL. A stable osteosarcoma model was obtained within 2 weeks, and significant lung metastasis occurred within 1–2 weeks. Mice in the experimental group (*n* = 10) were injected with MCA207 cells then subcutaneously injected with the Grp94/peptide vaccine (300 ng) or mHSP/peptide vaccine (30 µg). The vaccine injection was performed subcutaneously 1 day after MCA207 injection in the mice. The control group mice were injected with 0.2 mL of physiological saline (*n* = 10). Seventy-five mice were divided into 5 different groups (*n* = 15 each): the control group; Grp94/peptide group; mHSP/peptide group; PD-L1 inhibitor group; and mHSP/peptide plus PD-L1 inhibitor group. The mHSP/peptide sarcoma vaccine was administered at a therapeutic dose of 30 µg, and the Grp94/peptide sarcoma vaccine was administered at a therapeutic dose of 300 ng. The quantity of vaccine injected was based on our previous study [15, 16]. The animal experiments lasted 3–4 weeks. The growth of mouse tumors was measured from day 7, and the interval of measured time was 3 days. The vaccine effect was evaluated using a tumor growth curve and mouse survival time. The tumor transverse diameter (*A*) and long diameter (*B*) were measured every 3 days using a caliper, and the volume (*V*) was calculated by the formula *V* = (*A*2*B*/2). For the survival experiments, mice with tumorigenesis but no growth or tumor disappearance after treatment were considered therapeutically alive. Dead and live mice were counted continuously for 3 months.

### Bioinformatics for PD-L1 expression and osteosarcoma lung metastasis pathway analysis

Bioinformatics is widely used in cancer metastasis and the discovery of key proteins and signaling pathways. We performed a series of experiments to determine PD-L1 expression on sarcoma cell surfaces with IFN-γ.

To determine sarcoma cell surface PD-L1 expression, we downloaded the CD274 (PD-L1) expression dataset for cancer cell lines from the Cancer Cell Line Encyclopedia (CCLE: http://ualcan.path.uab.edu/index.html) [24]. The CCLE contains 1064 cell lines from 40 tissue sources, including 29 bone-derived tumor cell lines. The degree of CD274 expression in skeletal tumors was determined by comparing the mean value of CD274 expression in bone tumors with the mean values in other cell lines. This process was performed in GraphPad Prism. We also determined the signaling pathway of sarcoma metastasis-related PD-L1 expression on the cell surface. We downloaded the GSE85537 dataset from the Gene Expression Omnibus (GEO) database using the keyword “osteosarcoma metastasis” (Zhang W, Zhu J, unpublished data, 2016). Downloaded data files were processed using the R package application, which included the rejection of nonconforming data, data conversion, calibration, standardization and log2 conversion. We used the limma package for differential gene expression analysis to identify differentially expressed genes (DEGs). The DAVID database performs a Kyoto Encyclopedia of Genes and Genomes (KEGG) pathway analysis of DEGs online. We analyzed the expression of PD-L1 in different tumor cell lines and identified the primary KEGG pathway from the osteosarcoma lung metastasis microarray. A *P* value < 0.05 was considered statistically significant. We tested the results using Western blot (WB) experiments and histological staining, as described previously.

### X-ray and CT imaging evaluation of mice

After injection of MCA207 cells into the mice for the sarcoma model, subcutaneous injections of the vaccine and the PD-L1 immunological checkpoint inhibitor (Merck Company) were performed. Five mice were killed at 14 and 28 days and imaged using a small animal X-ray machine. The destruction of bone was evaluated using X-ray imaging. In another experiment, five mice from different treatment groups were anesthetized with 100 µL 5% pentobarbital at 28 days and evaluated using computed tomography (CT) imaging. Lung CT images of the five mice were acquired using micro-CT under the control of pentane gas. The data obtained were three-dimensionally (3D) reconstructed and analyzed in Mimics software to assess the size of the lung metastases in the treatment and control groups. Every lung specimen included 50 CT images, which were integrated in Mimics software. We marked low density as a bronchus or an alveolus and high density as a location of sarcoma lung metastasis. After the image integration process, we evaluated the integrated images using the ImageJ application.

### Statistics

Paired *t* tests and Student’s *t* tests were used for immunohistochemistry and ELISA results analyses. Survival was analyzed using GraphPad Prism 5 software (GraphPad Software, San Diego, CA, USA). One-way ANOVA was used to analyze tumor volume in mouse osteosarcoma. Two-way ANOVA was used to analyze tumor volume in antisarcoma cytokine experiments. *P* values < 0.05 were considered significant. *P < 0.05 **P < 0.01, ***P < 0.001.

## Results

### Expression of HSP70, HSP90 and Grp94 in MCA207 sarcoma cells and sarcoma tissue

**Fig. 2:**
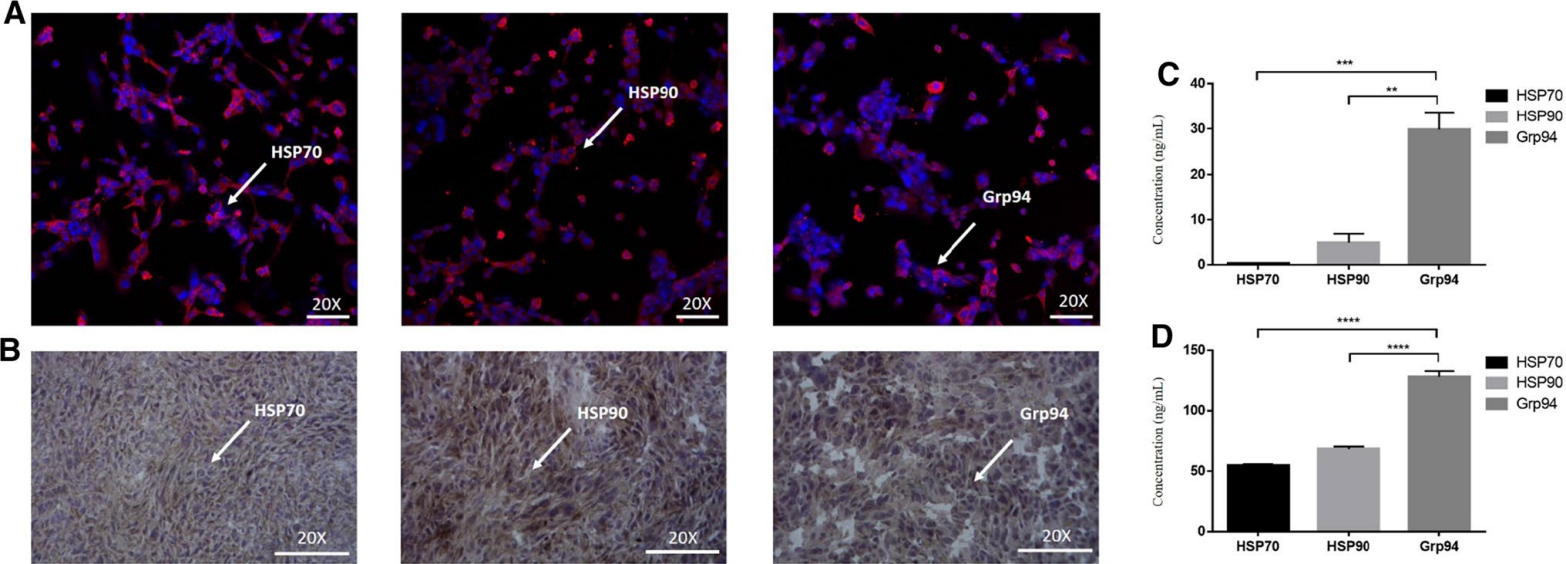
Expression and quantitative detection of HSP70, HSP90 and Grp94 in tumor cells and tumor tissues. The fluorescence microscope photos (20 ×) show HSP70, HSP90 and Grp94 expression on sarcoma cells (**a**). The immunohistochemical photos (20 ×) show HSP70, HSP90 and Grp94 expression in sarcoma tissue (**b**). The white arrow indicates a cell expressing HSP. ELISAs were used to quantify HSP in sarcoma cells (**c**) and sarcoma tissue (**d**) to determine the subtype of HSP for mass preparation. *P < 0.05 **P < 0.01, ***P < 0.001

Immunofluorescence staining showed that HSP70, HSP90, and Grp94 were stably expressed in MCA207 cells (Fig. 2a) and sarcoma tissues (Fig. 2b). The quantities of these three subtypes were measured separately in MCA207 cells (Fig. 2c) and sarcomas (Fig. 2d) (Tables 1, 2). The experimental results indicated that the Grp94 content in MCA207 cells and sarcomas was much higher than HSP70 and HSP90 contents. These results suggest that the high Grp94 level is a potential single vaccine component to compare with the mHSP/peptide. To demonstrate the treatment effect of mHSP/peptide, we used a high content of Grp94/peptide as a comparison group.

**Table 1:** The content of three subtype heat shock protein in MCA207 (x̅ ± s)

Subtype	Content
HSP70	0.2875 ± 0.03567
HSP90	5.008 ± 1.953
Grp94	29.81 ± 0

**Table 2:** The content of three subtype heat shock protein in sarcoma tissue (x̅ ± s)

Subtype	Content
HSP70	54.800 ± 0.9257
HSP90	68.055 ± 2.192
Grp94	127.70 ± 5.351

### Preparation of the mHSP/peptide vaccine and Grp94/peptide vaccine

**Fig. 3:**
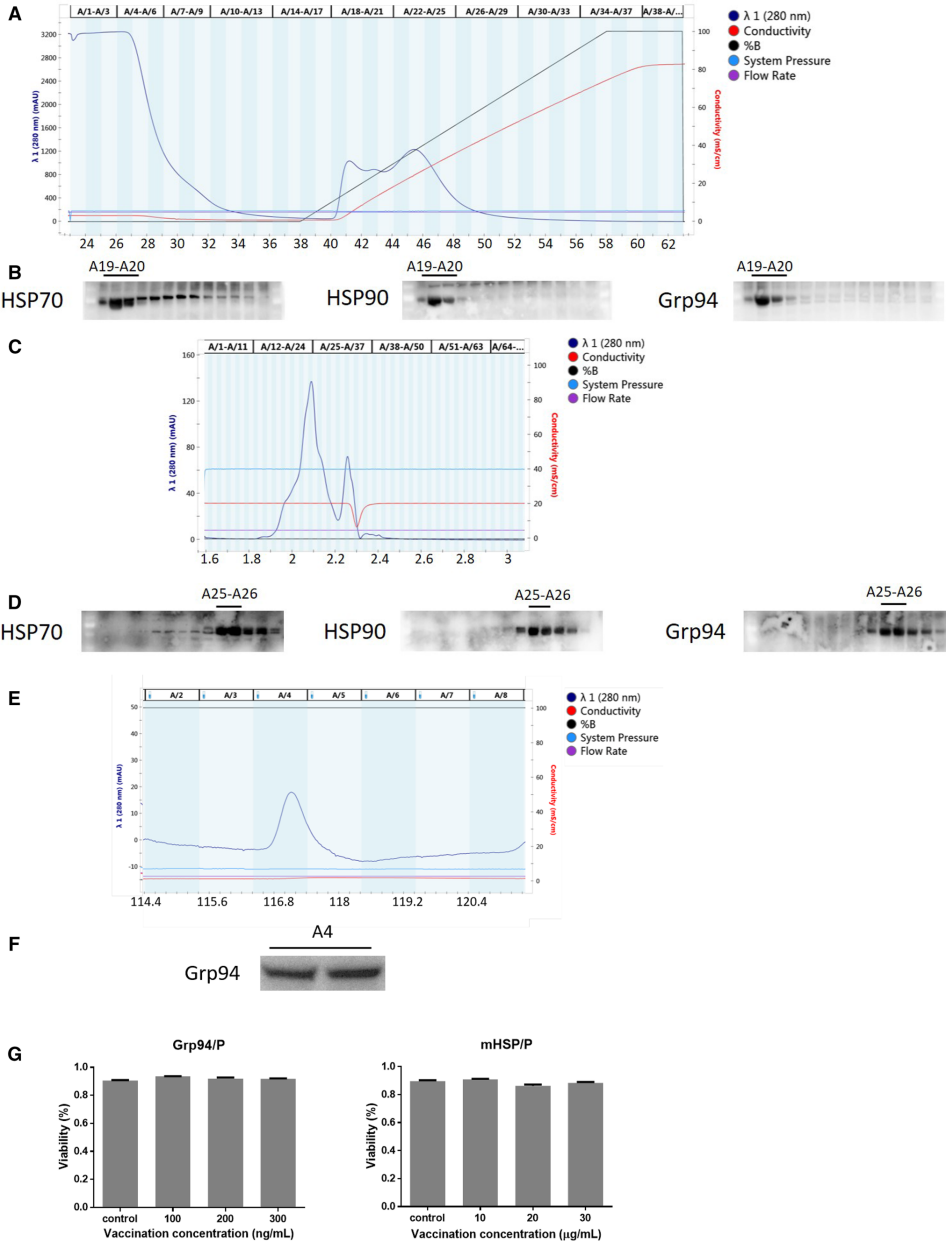
Preparation of the mHSP/peptide and Grp94/peptide vaccines and cytotoxicity tests. The ion-exchange chromatography process for protein purification (**a**) showing HSP70/peptide, HSP90/peptide and Grp94/peptide outflow fractions (**b**). The molecular chromatography process for mHSP/peptide preparation (**c**) showing mHSP/peptide outflow and mHSP/peptide, including HSP70/peptide, HSP90/peptide and Grp94/peptide (**d**). Immunoaffinity chromatography process (**e**) for Grp94/peptide preparation showing Grp94/peptide outflow fractions (**f**). Vaccine cytotoxicity tests indicated no cytotoxicity of the vaccines (**g**). *P < 0.05 **P < 0.01, ***P < 0.001

The specific progress of vaccine extraction was described in the text. Mouse tumor tissue was treated with a tissue lysate, centrifuged and loaded into an anion chromatography column (Fig. 3a). The mHSP/peptide sarcoma vaccine was prepared using molecular chromatography, and the Grp94/peptide vaccine was prepared using an affinity chromatography column. The mHSP/peptide preparation was subjected to anion chromatography, and fractions A17–A27 were collected as the first step (Fig. 3a). Western blot results showed that the HSP70, HSP90 and Grp94 proteins were present in the A19–A20 fractions (Fig. 3b). Substance from the A19–A20 fractions were purified using a molecular chromatography column, and the protein appeared primarily in the A15–A35 fractions (Fig. 3c). Western blots showed that HSP70 was present in the A25–A26 fractions, HSP90 was in the A25–A26 fractions, and Grp94 was in the A25–A26 fractions (Fig. 3d). We collected protein from the A25–A26 fractions as the mHSP/peptide vaccine. After the anion chromatography as the first step of mHSP/peptide preparation (Fig. 3a), we subsequently applied the substance to the affinity chromatography column to obtain the Grp94/peptide vaccine (Fig. 3e). The Grp94/peptide vaccine was obtained in the A4 fraction and tested using Western blotting (Fig. 3f).

### The mHSP/peptide vaccine and Grp94/peptide vaccine have no cytotoxicity

The cytotoxicities of the mHSP/peptide and Grp94/peptide vaccines were determined using flow cytometry. The cytotoxicity of mHSP/peptide was measured at different concentrations (10 µg/mL, 20 µg/mL, and 30 µg/mL), and no cytotoxicity was observed. The Grp94/peptide vaccine was tested at three concentrations (100 ng/mL, 200 ng/mL, and 300 ng/mL), and no cytotoxic effects were observed (*P * > 0.05) (Fig. 3g). We concluded that these vaccines did not have cytotoxicity and were suitable for in vivo injections.

### The mHSP/peptide vaccine has a stronger antitumor effect than the Grp94/peptide vaccine

**Fig. 4:**
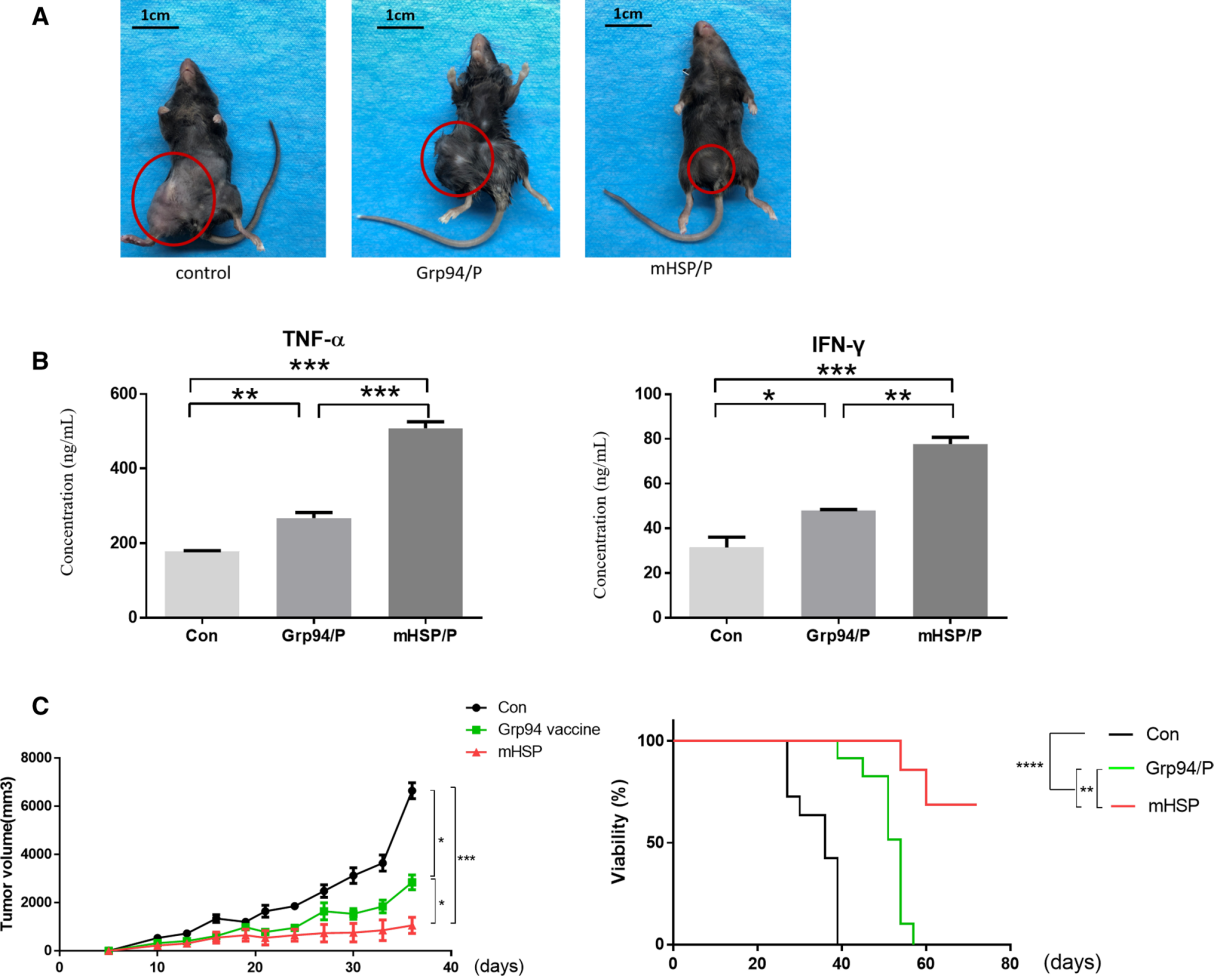
The mouse tumor growth is shown (**a**). In vivo vaccine experiments. mHSP/peptide and Grp94/peptide induce IFN-γ and TNF-α production, and mHSP/peptide is significantly better than the control and Grp94/peptide treatments (**b**). The mouse survival tests show that the mHSP/peptide and Grp94/peptide vaccines extend the survival time and delay tumor growth, and the experiments revealed that mHSP/peptide has more positive effects than the Grp94/peptide (**c**). *P < 0.05 **P < 0.01, ***P < 0.001

The antitumor effects of the vaccines were verified using antitumor experiments in mice (Fig. 4a). TNF-α and IFN-γ in tumor tissues were measured on the 14th day (Fig. 4b). Sarcoma size and survival rate were assessed at 3 months (Fig. 4c). ELISA experiments showed that the Grp94/peptide and mHSP/peptide stimulated the secretion of antitumor cytokines (Table 3), but the mHSP/peptide vaccine had a stronger effect on cytokine secretion than the Grp94/peptide vaccine. The Grp94/peptide (*n* = 10) and mHSP/peptide (*n* = 10) inhibited tumor growth in mice and prolonged the survival time. The mHSP/peptide exhibited a superior antisarcoma effect compared to the Grp94/peptide (Fig. 4b, *P < 0.05 **P < 0.01, ***P < 0.001).

**Table 3:** The Elisa experiment of IFN-γ and TNF-α measurement to ensure the antitumor effect of vaccines (x̅ ± s)

	Con	Grp94/peptide vaccine	mHSP/peptide vaccine
TNF-a	178.45 ± 3.19	267.90 ± 25.90	508.48 ± 29.98
IFN-γ	31.45 ± 8.14	48.06 ± 0.69	77.66 ± 5.20

### The cytokine IFN-γ increases PD-L1 expression on MCA207 sarcoma cells

**Fig. 5:**
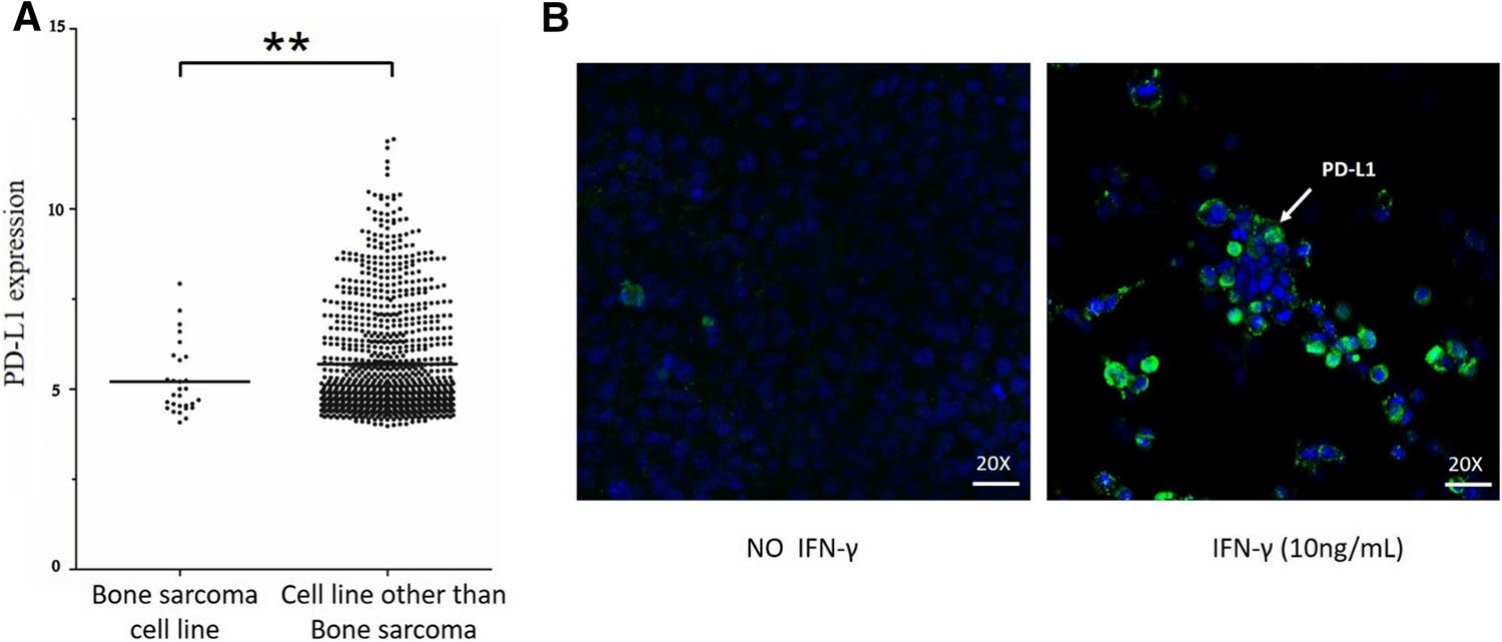
Expression of PD-L1 on the surface of osteosarcoma cells. Drawing from the online CCLE database, statistical analyses revealed that bone sarcoma cell lines in all stages show low expression of PD-L1 (**a**). Under IFN-γ stimulation, MCA207 sarcoma cells show a substantial increase in PD-L1 expression on the cell surface under a microscope (20 ×) (**b**). The white arrow points to a cell with HSP expression. *P < 0.05 **P < 0.01, ***P < 0.001

The expression of PD-L1 was assessed in 1064 cell lines on the CCLE website, and the average CD274 (PD-L1) expression was low in 29 sarcoma cell lines compared to tumor cell lines derived from tumor tissues, excluding sarcomas (Fig. 5a). For the in vitro experiments, IFN-γ (10 ng/mL) was added to stimulate MCA207 sarcoma cells for 24 h, and immunofluorescence staining showed that PD-L1 expression was higher than in cells without stimulation (Fig. 5b). These experiments showed that IFN-γ stimulated an increase in PD-L1 expression in sarcoma cells. PD-L1 is a ligand of PD-1 that enables immune escape to promote tumor metastasis. Although IFN-γ is an antitumor cytokine, it may stimulate PD-L1 expression on tumor cells. Therefore, the IFN-γ antitumor function is a stimulating factor of tumor metastasis and tumor immune escape. *P < 0.05 **P < 0.01, ***P < 0.001.

### IFN-γ stimulates PD-L1 expression to promote lung metastasis of osteosarcoma and active Akt expression

**Fig. 6:**
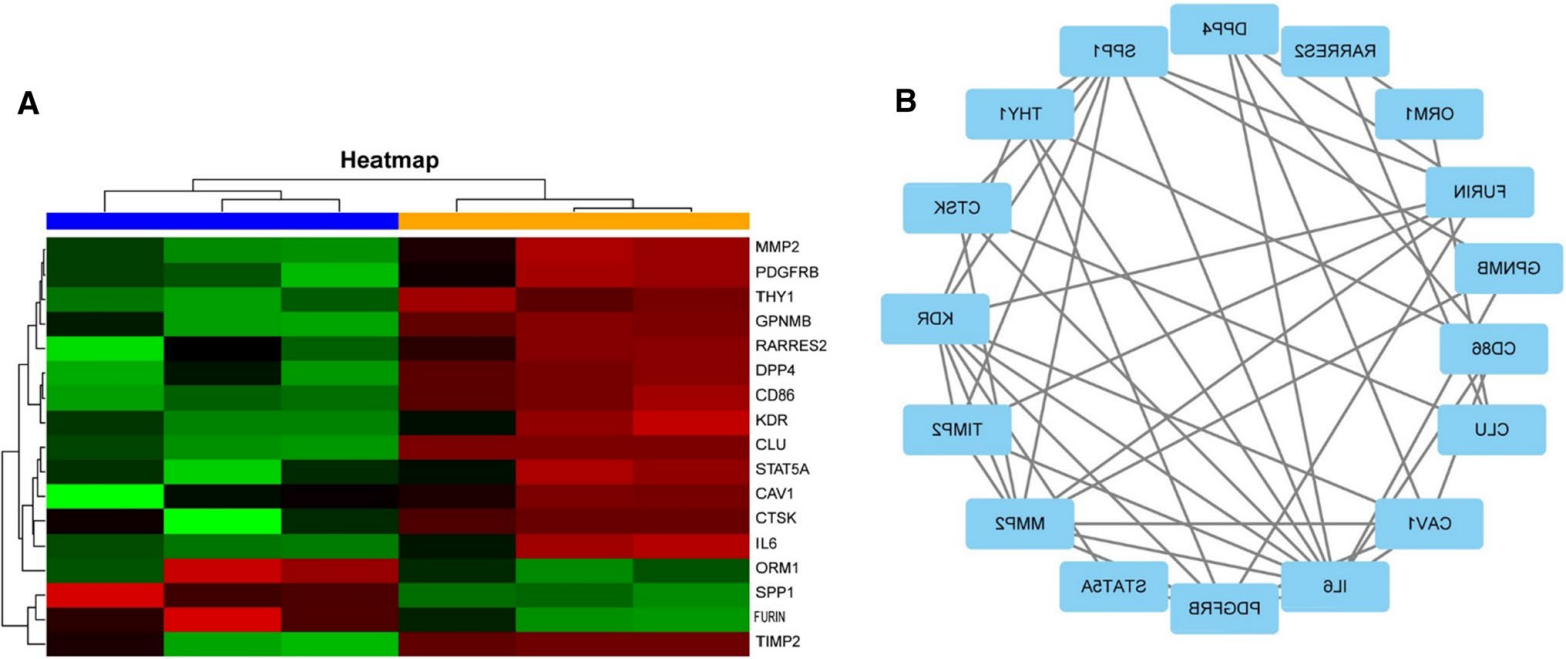
Bioinformatics analysis of the key genes between primary sarcoma and metastatic sarcoma and assessment of the signaling pathway of sarcoma metastasis. Heat map showing the top 17 differentially expressed genes in sarcoma metastasis (**a**). STRING chart showing the contacts of the 17 top genes, in which more nodes indicate more essentiality (**b**)

**Fig. 7:**
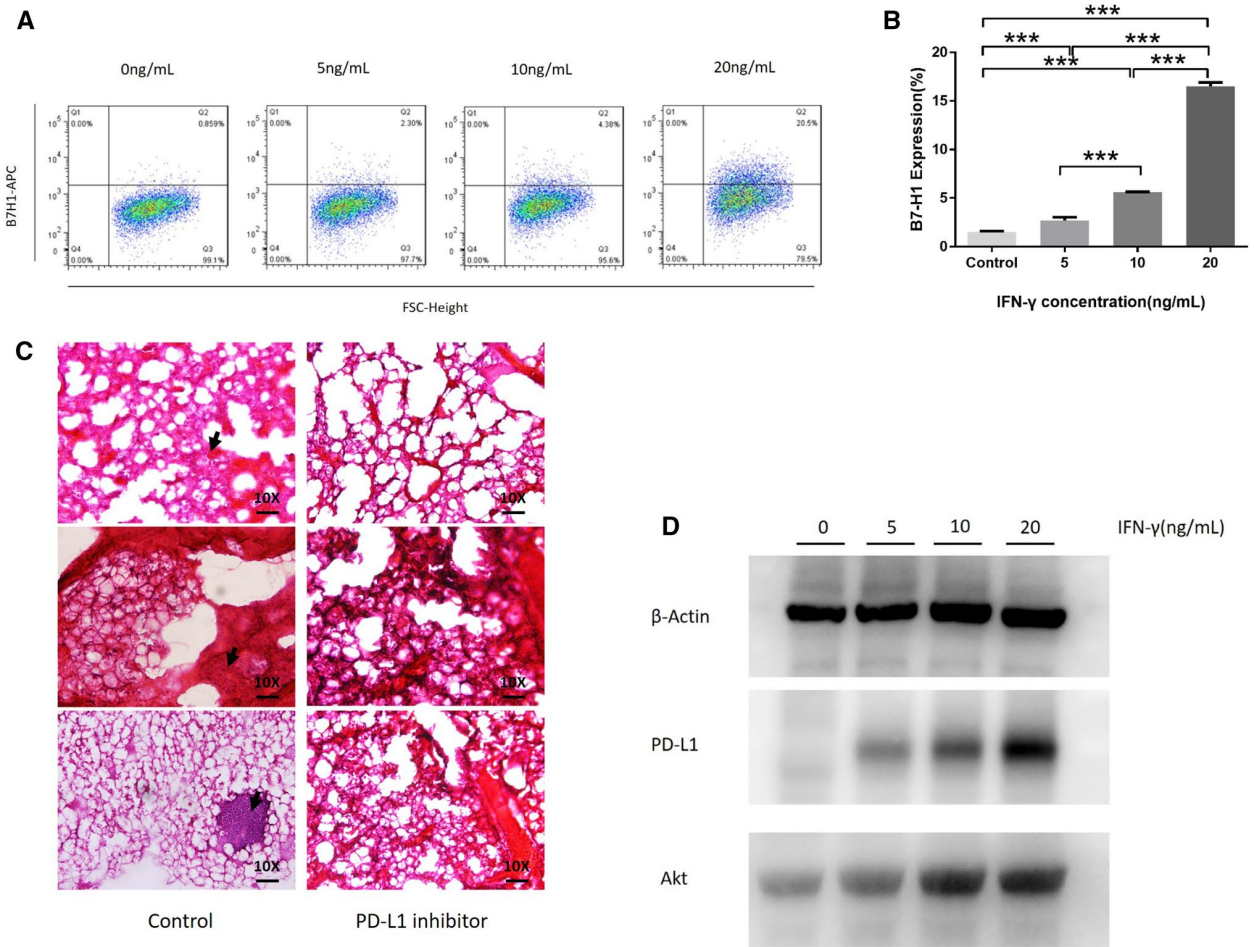
PD-L1 expression is associated with IFN-γ, and IFN-γ stimulates PD-L1 expression via the PI3K-AKT pathway. Flow cytometry (**a**) results showing PD-L1 expression changes with gradually increasing IFN-γ concentrations from 0 to 20 ng/mL (**b**). Sarcoma animal treatment with a PD-L1 inhibitor compared to the control treatment indicates that PD-L1 effectively hinders sarcoma metastasis, the black arrows point cells of metastasis (**c**). Western blot analysis of the PI3K-Akt pathway and PD-L1 expression with IFN-γ (0–20 ng/mL) stimulation indicates that PD-L1 and Akt expression increases with IFN-γ stimulation (**d**)

We used flow cytometry and showed that along with the concentration of IFN-γ increasing, IFN-γ stimulated PD-L1 expression. We then used bioinformatics to identify important genes and essential signaling pathways and verified the pathways using Western blotting and pathology hematoxylin and eosin (HE) staining. The GSE85537 data package was downloaded from the GEO database and contained a total of six samples, including three orthotopic osteosarcoma samples and three osteosarcoma lung metastasis samples. The data analyses of these samples revealed 137 key genes from the differential genes using topological analysis, and 17 key genes were marked in a heat map (Fig. 6a). These 17 key genes were selected using MCODE analysis based on the node number in the STRING analysis (Fig. 6b). KEGG was used on the DAVID website and indicated that the PI3K-Akt pathway may be the key pathway for lung metastasis of osteosarcoma (Table 4). We have verified the Akt expression increased along with the metastasis. PD-L1 (B7H1) expression on MCA207 cells increased with increasing concentrations of IFN-γ (0–20 ng/mL) (Fig. 7a, b). We used HE staining and found that a PD-L1 inhibitor successfully inhibited lung metastasis (Fig. 7c). Western blotting was used to verify the Akt expression in sarcoma metastasis. Akt and PD-L1 levels exhibited an upward trend, and the β-actin level remained constant (Fig. 7d). In summary, in vitro experiments showed that the increase in IFN-γ concentration activated the Akt expression and promoted the expression of PD-L1 on sarcoma cells. In support of this hypothesis, mice bearing sarcomas and treated with a PD-L1 inhibitor had no obvious lung tumor metastases on the 14th day (Fig. 7c) (*n* = 5). Lung metastasis of sarcomas is maybe associated with the PI3K-Akt pathway, and these results indicated that IFN-γ promoted it. We suggest that the addition of PD-L1 immunological checkpoint inhibitors will effectively inhibit this process, and the PI3K-Akt pathway maybe is one signaling pathway of sarcoma metastasis.

**Table 4:** Signaling pathway of sarcoma metastasis is searched by bioinformatics analysis and we look at the pathway of PI3K-Akt

KEGG	*P* value	Genes
Toll-like receptor signaling pathway	1.58*E *− 05	CTSK, CD86, IL6, SPP1
PI3K-Akt signaling pathway	0.001591921	IL6, PDGFRB, SPP1, KDR
Focal adhesion	0.003726435	CAV1, PDGFRB, SPP1, KDR
Cytokine–cytokine receptor interaction	0.005443912	IL6, PDGFRB, KDR
Rheumatoid arthritis	0.00944621	CTSK, CD86, IL6

### Immunotherapy for sarcomas has tremendous effects, and this process was assessed in a series of animal experiments, including X-ray and daily tracking measurements

**Fig. 8:**
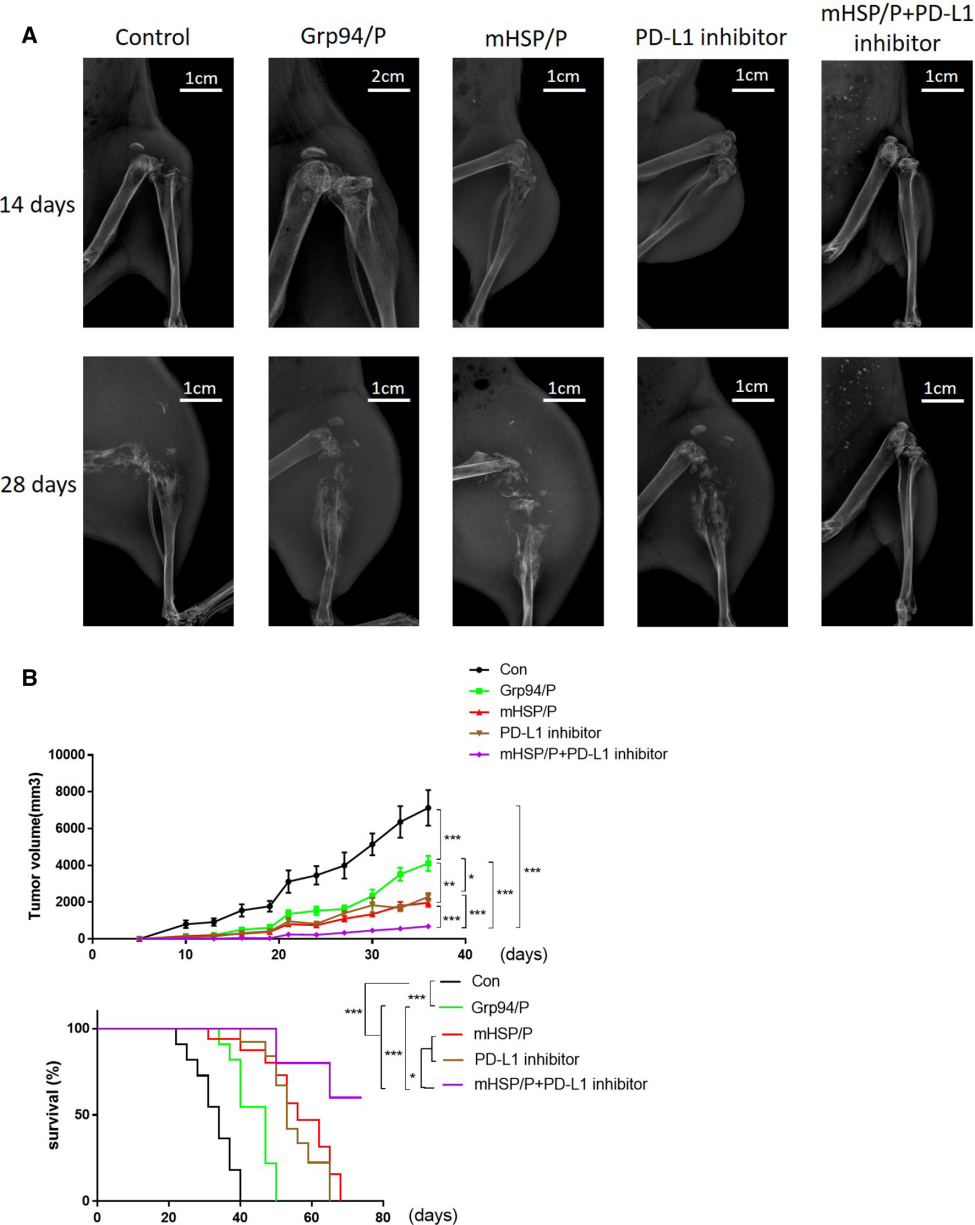
X-ray imaging of different treatments for sarcoma deterioration. We measured the tumor size and sarcoma progression and monitored the survival of mice bearing sarcoma over time. X-ray images show that combination therapy has more opportunities to prevent bone destruction by sarcoma than single treatments (**a**). Daily sarcoma size measurement and survival time results indicate that combination therapy delays sarcoma development and prolongs mouse survival (**b**). *P < 0.05, **P < 0.01, ***P < 0.001

Animal experiments were used to evaluate immunotherapy, and we divided the animals into five groups (*n* = 20). We segregated our experiments into two parts: one part was for X-ray evaluation, and the other part was for the tumor size and the survival rate over time. For the first part, we injected vaccines and checkpoint inhibitors into MCA207 cell-bearing mice, as described in the “Materials and methods”. The five groups were the control group, Grp94/peptide group, mHSP/peptide group, PD-L1 inhibitor group and mHSP/peptide plus PD-L1 inhibitor group. X-ray images revealed that immunotherapy combined with a checkpoint inhibitor resulted in less bone degeneration than the single treatments (Fig. 8a). We observed the five groups (*n* = 5) at the 14th and 28th days. There were no crucial differences between the five groups, and we hypothesized that there was no essential bone degeneration during these 2 weeks. However, we found significant destruction in the control and single treatment groups on the 28th day. The mHSP/peptide vaccine plus PD-L1 inhibitor inhibited sarcoma, and some mice were living with tumors. The difference is notable in the pictures (Fig. 8b). We hypothesized that combination therapy enhanced the antitumor effect and was more powerful than each individual component. In the second part, we evaluated the survival rate and tumor growth size in five groups of mice (*n* = 15). We found significant differences between the five groups. Single treatments and combination therapy inhibited deterioration at 2 months, but the single treatments were not as effective as the combination treatment. There were no significant differences between the PD-L1 inhibitor and mHSP/peptide vaccine groups (Fig. 8c). For the survival survey, the mHSP/peptide vaccine prolonged mouse survival beyond 3 months, but the other four treatments did not prolong survival. Pairwise comparisons found differences between the control and experimental groups. Differences existed between the combination therapy and single treatment groups, and the mHSP/peptide vaccine was more effective than the other treatments (Fig. 8c). In summary, we conclude that the mHSP/peptide vaccine plus a PD-L1 inhibitor may be an alternative sarcoma treatment.

### Combined immunotherapy stimulates the effects of antitumor cytokines

**Fig. 9:**
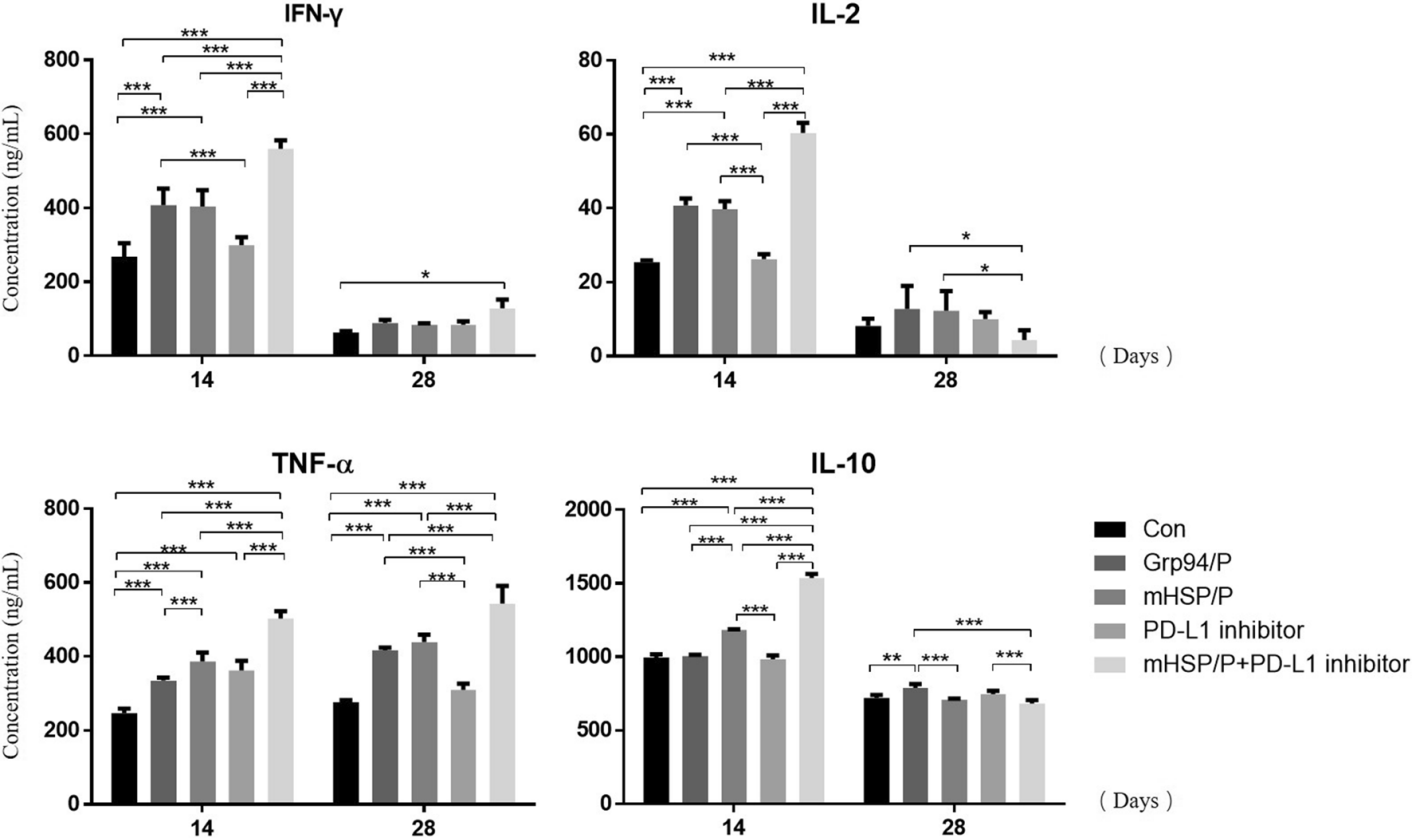
ELISA tests at the 14th and 28th days. These tests include IFN-γ, TNF-α, IL-2, and IL-10 ELISAs. *P < 0.05, **P < 0.01, ***P < 0.001

This experiment evaluated the effects of combination therapy and single therapy based on cytokine secretion using ELISA to quantify IFN-γ, TNF-α, IL-2, and IL-10 which is a negative immunoregulatory factor, in tumor tissues. The cytokines in the tumor tissue were quantitatively determined on the 14th and 28th days. The results are shown in Fig. 9, Tables 5 and 6. The IFN-γ, TNF-α, IL-2 and IL-10 levels on the 14th day showed that the combined immunotherapy induced the production of cytokines to promote antitumor effects. The experimental group exhibited greater antitumor effects than the control group. The single PD-L1 inhibitor treatment did not have as large an effect as the Grp94/peptide and mHSP/peptide treatments. On the 28th day, the effect of combination immunotherapy on the secretion of antitumor cytokines was significantly reduced. IL-10 is a negative immunoregulatory cytokine for tumor immune escape, but it showed a trend of increased expression in these experiments. In summary, we concluded that four experimental groups showed cytokine stimulation, but these effects of the vaccines gradually vanished over time, but IL-10, as an immunoregulatory cytokine, remained in the tumor environment to inhibit antitumor function.

**Table 5:** The cytokines secretion of five groups on 14th day (x̅ ± s)

	Con	Grp94/peptide vaccine	mHSP/peptide vaccine	PD-L1 inhibitor	mHSP/peptide vaccine + PD-L1 inhibitor
IFN-γ	268.58 ± 36.712	407.58 ± 45.01	404.38 ± 44.11	299.50 ± 21.62	560.47 ± 22.70
IL-2	25.34 ± 0.58	40.76 ± 1.84	39.69 ± 2.22	26.25 ± 1.33	60.40 ± 2.67
IL-10	996.65 ± 21.06	1004.08 ± 9.63	1182.08 ± 5.56	983.68 ± 25.69	1536.23 ± 28.55
TNF-a	246.36 ± 12.40	334.81 ± 8.09	386.64 ± 23.98	362.19 ± 26.39	502.41 ± 20.01

**Table 6:** The cytokines secretion of five groups on 28th day (x̅ ± s)

	Con	Grp94/peptide vaccine	mHSP/peptide vaccine	PD-L1 inhibitor	mHSP/peptide vaccine + PD-L1 inhibitor
IFN-γ	63.77 ± 3.34	88.98 ± 8.76	84.39 ± 3.68	84.23 ± 9.62	128.69 ± 23.43
IL-2	8.15 ± 1.97	12.77 ± 6.19	12.22 ± 5.33	10.05 ± 1.87	4.32 ± 2.67
IL-10	720.38 ± 20.06	788.99 ± 25.08	707.40 ± 8.50	746.34 ± 22.48	683.30 ± 23.16
TNF-a	276.00 ± 5.57	416.23 ± 7.16	439.12 ± 19.73	309.46 ± 17.08	542.74 ± 47.96

### The mHSP/peptide vaccine plus a PD-L1 inhibitor stimulates the expression of CD4 + and CD8 + T cells and inhibits the expression of Foxp3 + T cells

**Fig. 10:**
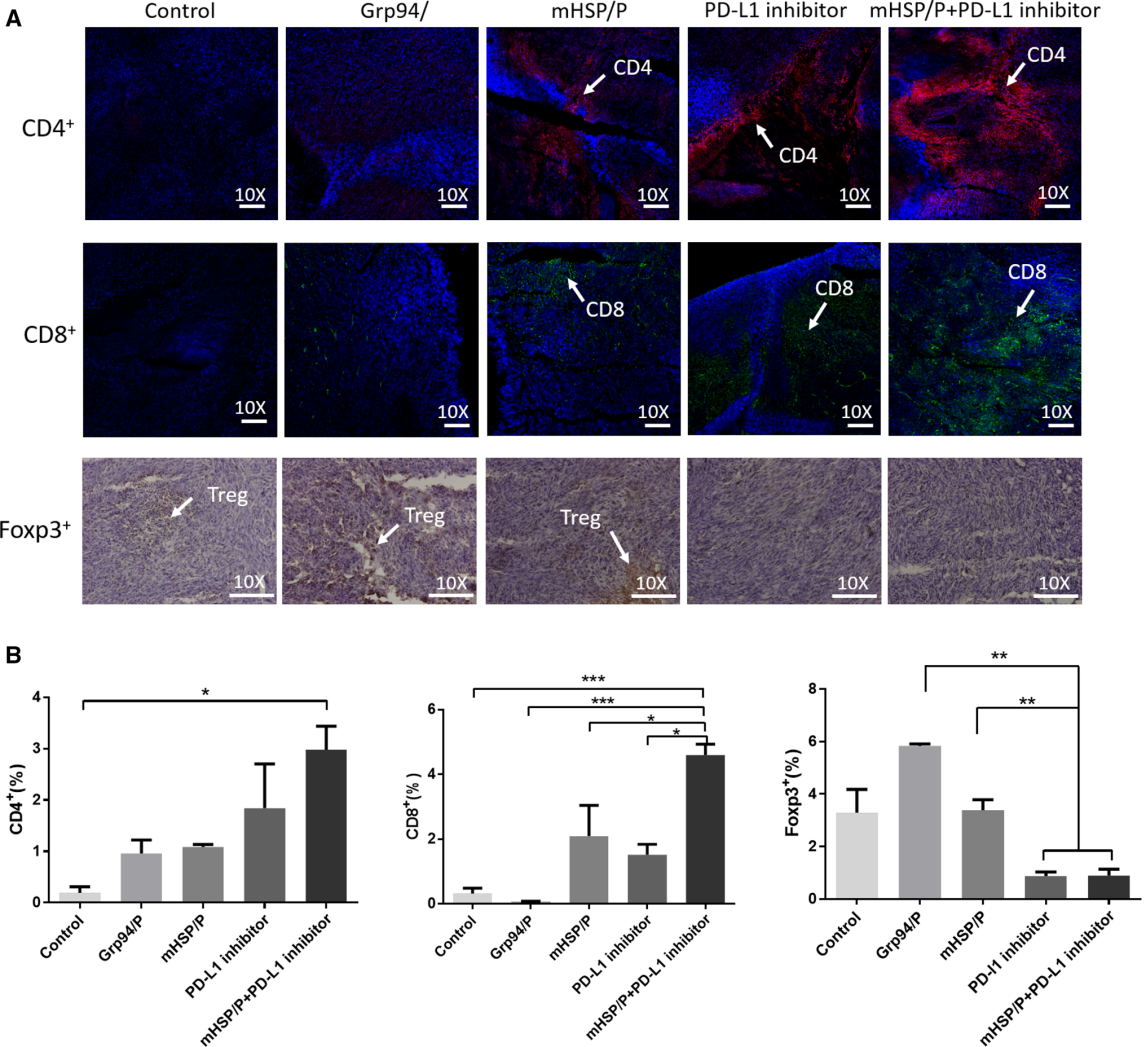
Three different immune cells (CD4 + T cells, CD8 + T cells, and Foxp3 T cells), signed using white arrows, infiltrate in sarcoma tissue are measured for treatment evaluation. Under a microscope (10 ×), we observe that combined therapy induces more CD4 + T and CD8 + T cells than single treatment, but the Foxp3 T (Treg) cells appear less abundant than with the single treatments (**a**). We analyse the treatment groups to determine the effects of combination treatment (**b**)

As important participants in innate immunity and adaptive immune responses, T cells play important roles in the prevention and treatment of sarcomas. We measured CD4 + and CD8 + T cells as antitumor immune cells and Tregs, which have immunomodulatory effects, and found that combination immunotherapy stimulated CD4 + and CD8 + T cells and prevented the infiltration of Tregs. Immunohistochemical staining of tumors in each group of mice (*n* = 5) indicated that the combination treatment of mHSP/peptide vaccine plus a PD-L1 inhibitor promoted an increase in CD8 + and CD4 + T cells but hindered Treg proliferation. The other experimental groups showed that a single vaccine promoted the infiltration of CD8 + and CD4 + T cells but did not suppress Treg cells. We also found that a PD-L1 inhibitor inhibited the expression of Foxp3 + but had less effect on CD4 + and CD8 + T cells (Fig. 10a). As a negative regulation of immune T cells, Tregs promote sarcoma metastasis and immune escape, and combination therapy hindered these effects of metastasis and immune escape. In summary, the combined immunotherapy of the mHSP/peptide plus a PD-L1 inhibitor inhibited the negative regulation of Tregs and stimulated antitumor T cells (Fig. 10b).

### The combination therapy successfully inhibits lung metastasis as evaluated on CT images

**Fig. 11:**
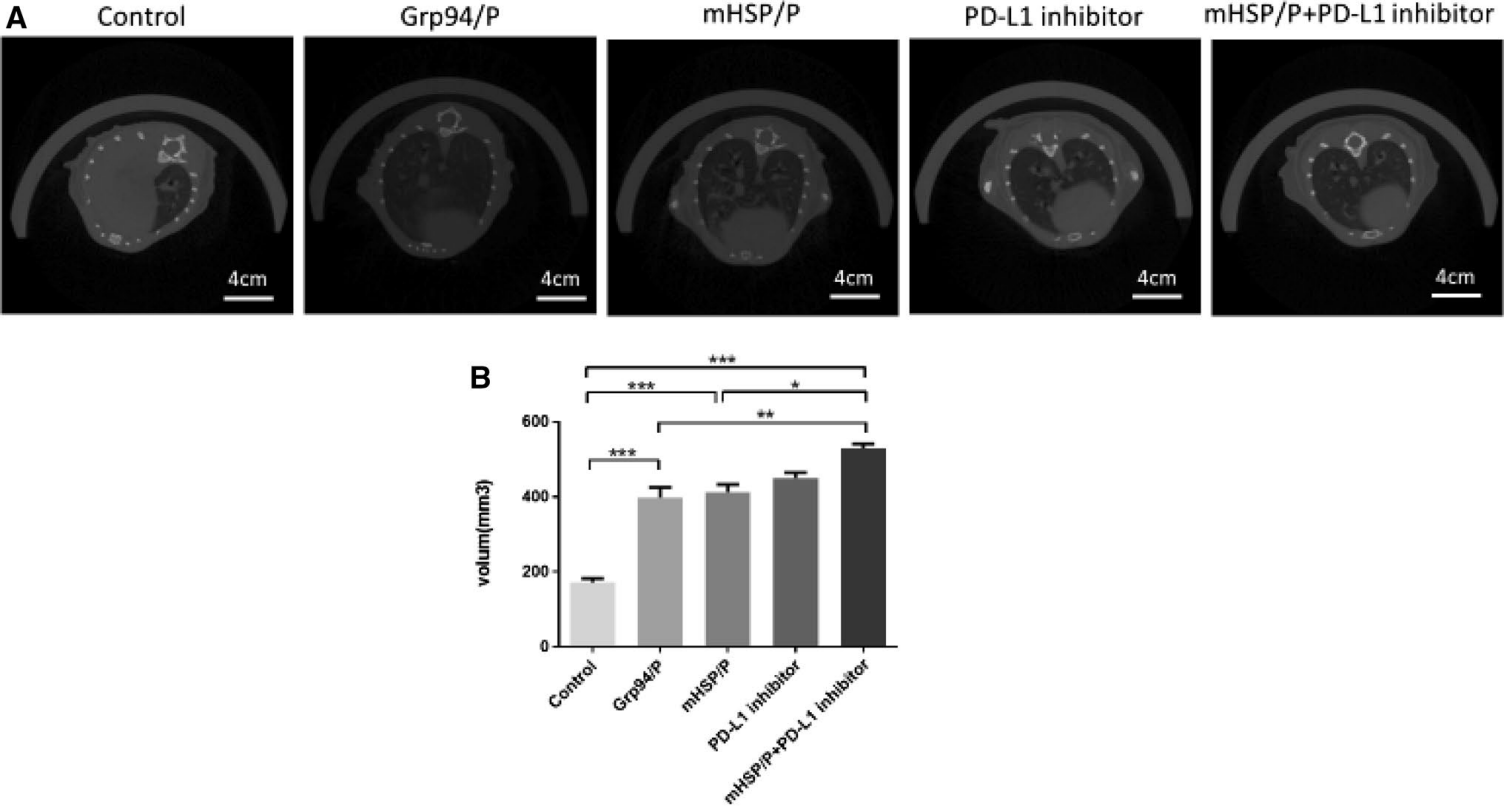
Comparison of the different treatment groups with the control group indicate that combined therapy affect sarcoma metastasis. CT images show sarcoma metastasis under different treatments (**a**), and the 3D reconstruction method is used to evaluate lung metastasis (**b**)

CT images were used to evaluate differences between the control group and the experimental treatment groups (*n* = 5). The lung volume was calculated after 3D reconstruction using Mimics software (Materialise Company; Belgium). The calculation showed that the lung volume of the blank control group was occupied by metastatic sarcomas. We used high-density shadows in CT images to represent sarcomas and low-density shadows to represent alveolar tissue. We concluded that the experimental treatments hindered the deterioration of the lung, and combination therapy successfully stopped sarcoma progression and metastasis (Fig. 11).

## Discussion

Immunotherapy directly or indirectly activates the immune system and results in the lysis of tumor cells or the secretion of cytokines that destroy tumor cells to achieve antitumor effects [25]. Tumor vaccines, as a tumor immunotherapy [26, 27], achieve antitumor effects in vivo by inducing a strong immune response against sarcomas. With research progress on the tumor microenvironment, immunotherapy for sarcomas is increasingly applied. The tumor immune microenvironment plays an important role in influencing the antitumor effect of immune cells. Tumor vaccines stimulate the body to produce an antitumor immune response, but the negative regulation of immunity is also important for sarcoma elimination. For example, immune cells entering the tumor microenvironment may change their markers to become more immunomodulatory [28]. We isolated tumor tissue and prepared an mHSP/peptide vaccine using chromatography. Our experimental study of the antitumor mHSP/peptide found that the production of the antitumor cytokine IFN-γ stimulated the expression of PD-L1 on tumor cells, which resulted in immune escape and lung metastasis of tumor cells. The addition of a PD-L1 inhibitor effectively inhibited the occurrence of tumor cell immune escape events. The combination of vaccines and checkpoint inhibitors represents a new treatment for sarcomas.

The HSP vaccine prepared using the chromatographic method was derived from the tumor tissue of C57BL/6 mice [29], and the vaccine is different from traditional HSP vaccines, including HSP70 [30–32], HSP90 [8, 33], and Grp94 [34]. These subtypes were used as tumor vaccines in previous experimental studies. We also prepared a single component Grp94/peptide vaccine. The antitumor effect of the Grp94/peptide vaccine was not as strong as the mHSP/peptide, which indicated that the mHSP/peptide vaccine was much better than the Grp94/peptide vaccine. We demonstrated that the mixed vaccine delayed tumor growth in animal experiments, increased the survival rate and increased the expression of the antitumor cytokines IFN-γ and TNF-α. We hypothesize that the mHSP/peptide sarcoma vaccine carries more molecular peptides as tumor antigens than the single Grp94/peptide sarcoma vaccine, which provides more targets for antigen-presenting cells (APCs). The vaccine was prepared in our laboratory. HSPs are continuously expressed as chaperone proteins in tumor cells, and protein loss during purification and vaccine preparation cannot be effectively avoided. The amount of vaccine prepared is often the greatest problem of these experiments. The mHSP/peptide vaccine successfully overcomes this shortcoming. However, there is another problem; the concentration of the Grp94/peptide sarcoma vaccine that was used was not necessarily the optimal concentration to stimulate an effective immune response because of the problem of quantifying the Grp94/peptide.

IFN-γ was used as an antitumor cytokine in previous studies. However, elevated IFN-γ concentrations in the local microenvironment of tumors affect the antitumor ability of T lymphocytes in the tumor microenvironment [35], and this process is closely related to changes in PD-L1 expression on sarcoma cells. With the increase in IFN-γ content in the local microenvironment, the expression of PD-L1 on the surface of tumor cells also showed an increasing trend [36, 37]. We downloaded information on the expression of PD-L1 in sarcoma cell lines from the CCLE and found that osteosarcoma cells generally have low expression of PD-L1, but PD-L1 expression increased with the increased expression of IFN-γ. This result supports our hypothesis that the mHSP/peptide vaccine also had an antitumor effect and caused tumor cell immune escape via IFN-γ secretion. The data analyses of the gene chip experiment (GSE85537) for orthotopic osteosarcoma and lung metastases revealed that the PI3K-AKT pathway was important in the process of orthotopic osteosarcoma. AKT and PD-L1 expression also increased with the increase in IFN-γ levels. Tumor metastasis was significantly inhibited after the addition of the PD-L1 inhibitor. The relationship between PD-L1 and the PI3K-AKT pathway was revealed in a previous study [37]. We further verified that PD-L1 promoted the immune escape of tumor cells and lung metastasis of osteosarcoma via the PI3K-AKT pathway. Although we used different methodologies to support this hypnosis and achieve success, additional research is needed to verify it in more numerous subjects.

To avoid PD-L1-mediated immune escape while achieving the vaccine-induced antitumor immune response, we increased the antitumor effect of the vaccine by adding a PD-L1 immunological checkpoint inhibitor. PD-L1 inhibitors and PD-1 inhibitors improve the antitumor effect of sarcoma vaccines [38].

We evaluated the therapeutic effects of combined immunotherapy in vivo and in vitro. The effective time of the vaccine-promoted antitumor cytokines in the mice was longer than 2 weeks but less than 4 weeks. Achieving a slow release of the tumor vaccine to ensure effective and continuous immune-stimulating ability is worth considering in the future. Current studies on sustained-release vaccines were reported in the literature [10, 39], but experiments and protocols for mHSP vaccines have not been developed.

We also measured a variety of immune cells in the tumors, including CD4 + T cells, CD8 + T cells, and Tregs [40–42]. CD4 + and CD8 + T cells kill tumor cells via specific immune responses, especially CD8 + T cells [43]. The combination immunotherapy promoted the invasion of immune cells into the tumor microenvironment to some extent. Therefore, the immune negative regulatory cells Tregs were also tested, and the combined immunotherapy reduced Tregs in the tumor microenvironment. Combined immunotherapy reduced the invasion of negative immune regulatory cells and enhanced the infiltration of killer lymphocytes. Future studies should consider other lymphocytes, such as regulatory B cells (Bregs), to verify our results.

HSPs are widely expressed in the body, and their content in tumor tissues is elevated. Preparation of the mHSP/peptide vaccine using autologous tumor tissue avoids the lack of a protein source and enables the acquisition of peptides containing autologous tumor tissue antigens. mHSP/peptide has a promising future for clinical transformation. In addition, PD-L1 immunosuppressants effectively inhibit the PD-L1 induction of IFN-γ on tumor cells.

## Conclusion

The present study successfully prepared an mHSP/peptide vaccine and demonstrated that the mHSP/peptide may be safely used in vivo in mice without cell toxicity. In vitro and in vivo experiments demonstrated that the mHSP/peptide sarcoma vaccine had stronger antitumor ability than the single Grp94/peptide vaccine and single PD-L1 inhibitor. The study found that mHSP/peptide stimulated IFN-γ production, which promoted PD-L1 expression on tumor cells, and PD-L1 overexpression led to sarcoma metastasis. Therefore, we designed a combination therapy of mHSP/peptide plus a PD-L1 inhibitor, and this therapy inhibited tumor progression. In summary, we developed an mHSP/peptide vaccine against osteosarcoma and elucidated the antitumor mechanism of its use in combination with PD-L1 inhibitors. This combined treatment may be a new alternative treatment for sarcoma.

## Data Availability

The data used in this article may be obtained from the first author on reasonable request.

## Abbreviations

PD-L1: Programmed cell death protein ligand 1
mHSP/peptide: Mixed heat shock protein/peptide sarcoma vaccine
HSPs: Heat shock proteins
CT: Computed tomography
OS: Osteosarcoma
ER: Endoplasmic reticulum
SDS-PAGE: Sodium dodecyl sulfate–polyacrylamide gel electrophoresis gel
PVDF: Polyvinylidene fluoride
HRP: Horseradish peroxidase
CCLE: Cancer Cell Line Encyclopedia
GEO: Gene Expression Omnibus
